# The impact of the Paris terrorist attacks on the mental health of resident physicians

**DOI:** 10.1186/s12888-019-2058-y

**Published:** 2019-02-21

**Authors:** Jules Gregory, Jean de Lepinau, Ariane de Buyer, Nicolas Delanoy, Olivier Mir, Raphaël Gaillard

**Affiliations:** 1 Syndicat des Internes des Hôpitaux de Paris (SIHP), 17 rue du Fer à Moulin, 75005 Paris, France; 2 Department of Cancer Medicine, Gustave Roussy Cancer Campus, Université Paris-Saclay, Villejuif, France; 3 Department of Psychiatry, Service Hospitalo-Universitaire, Centre Hospitalier Sainte Anne, Université Paris Descartes, Sorbonne Paris Cité, Faculté de Médecine Paris Descartes, Paris, France

## Abstract

**Background:**

On November 13, 2015, terrorist attacks took place in Paris. One hundred and twenty-nine people were immediately killed and 302 needed emergency care. Many resident physicians were on the front line of the medical response. Our aim was to report the frequency of symptoms of post-traumatic stress disorder (PTSD), anxiety and depression among resident physicians after the Paris terrorist attacks.

**Methods:**

Anonymous questionnaires, including the Impact of Event Scale- Revised (IES-R) and the Hospital Anxiety and Depression Scale (HADS), were emailed two months after the attacks to 2413 Parisian resident physicians. Exposure to the attacks was defined as having direct clinical contact with one of the victims up to one week after the attacks, being one of the victims, or having one among close relatives.

**Results:**

The questionnaire was completed by 680 (28.2%) residents. Eighty-four (12.4%) reported symptoms of PTSD (IES-R ≥ 33), 76 (11.2%) reported symptoms of anxiety (HADS anxiety score > 10) and 16 (2.4%) reported symptoms of depression (HADS depression score > 10). Exposed residents had higher IES-R scores than non-exposed residents (18.8 ± 16.6 versus 14.2 ± 12.0, *p* = 0.001), and 40 (18.5%) of them reported symptoms of PTSD, compared to 44 (9.5%) of the non-exposed residents (*p* = 0.001).

**Conclusions:**

There was a high frequency of symptoms of mental distress among our respondents. Dedicated screening and care strategies must be considered in the event of new attacks.

**Electronic supplementary material:**

The online version of this article (10.1186/s12888-019-2058-y) contains supplementary material, which is available to authorized users.

## Background

On the evening of Friday, November 13, 2015, terrorist attacks took place in six different locations in and around Paris. One hundred and twenty-nine people were immediately killed and 302 needed emergency care [1]. Health professionals from Parisian hospitals played a massive role in caring for the victims [2].

Resident physicians, who are junior doctors completing their training in any specialization, were on the front line of the medical response to the Paris attacks, either performing pre-hospital care at the site of the massacre or taking charge of the injured at hospital, likely confronted for the first time by such a tragic situation.

Being a first responder or survivor of a terrorist attack can induce serious mental health problems. These may occur post exposure because of a close relationship with a victim or repeated confrontations with narratives of the event, provided they contain enough shock or terror. Breslau et al. [3] highlights that inter-personal trauma, including intentional violence between human beings, is more likely to cause psychiatric consequences than indirect trauma. The prevalence of mental health problems among persons involved in rescue efforts after a human-made disaster is significantly higher than in the general population, but lower than among direct victims. After the “September 11th attacks”, these outcomes were particularly studied among social workers, highlighting the prevalence of traumatic stress among the professionals caring for victims [4, 5]. The occurrence of traumatic stress is linked to interacting with and identifying with the victims [6]. It is associated with anxiety, depression and post-traumatic stress disorder (PTSD). The risk of traumatic stress is especially high among the professionals caring for victims, as they are an integral part of the community under attack. Their cognitive patterns and sense of personal safety are compromised, reducing their emotional distance from the victims.

Medical workers are at a higher risk of developing mental health disorders, and a number of studies suggest that resident physicians are particularly vulnerable. In a recent meta-analysis, Mata et al. [7] estimated the prevalence of depression or depressive symptoms among all specialties of residents at 28.8%, ranging from 20.9 to 43.2%. With regards to stress at work, anxiety rates among residents have been reported to range from 8 to 40% [8–11]. Rates of work-related PTSD among residents are reported to be around 10% [12, 13]. Furthermore, residents lack experience and have no formal preparation for exceptional situations, such as terror attacks, yet they play an important role in the care pathways of victims. Therefore, they may be a higher risk of developing post-traumatic stress difficulties.

There are no published studies that describe the effect of terrorist attacks on resident physicians. Given the current terror threats, we aimed to emphasize the psychological consequences of these attacks, in order to encourage improved care for residents and immediate psychiatric help in the case of future attacks.

The objective of our study was to describe the frequency of symptoms of PTSD, anxiety and depression among Parisian resident physicians after the terrorist attacks on November 13, 2015.

## Methods

### Conduct of the study

This cross-sectional study was based on a survey of resident physicians working in the hospitals of the Assistance-Publique des Hôpitaux de Paris (APHP) (approximately 3600 residents) in November 2015 and affiliated with the Parisian resident association (Syndicat des Internes des Hôpitaux de Paris — SIHP) (2413 residents). In January 2016, a single email containing a link to a web-based, anonymous, self-reporting questionnaire Additional file 1 was sent to all 2413 SIHP affiliated resident physicians. No particular survey mailing strategy was used. The questionnaire was available for one month and could only be completed once. Only complete questionnaires were analyzed.

As we were concerned about the psychological distress of our colleagues but were unable to contact them in order to preserve anonymity, we added the contact details (by email or phone) of a dedicated support number to the questionnaire. This support number, affiliated to the SIHP and managed by psychiatry residents, offered free appointments with a dedicated senior psychiatrist in less than 24 h and if necessary, special care in a Parisian psychiatric department. Due to anonymity, we have no data on the frequency of calls to that support number. The study protocol was approved by one of the APHP Ethics Committee.

### Outcome variables

Our study focused on three main outcomes: PTSD, anxiety, and depression. Resident physicians were asked to complete the Impact of Event Scale- Revised (IES-R) [14, 15] and the Hospital Anxiety and Depression Scale (HADS) [16].

The IES-R is one of the most commonly used scales to assess the subjective distress caused by traumatic events [17, 18]. It is based on the DSM-4 criteria for PTSD. It is a self-assessment questionnaire that includes 22 items measuring intrusion, avoidance and neurovegetative hyperactivity. Each question is scored on a Likert scale from 0 to 4. Respondents are asked to indicate how often they experienced each of the 22 items in the last seven days (0 = Not at all; 1 = A little bit; 2 = Moderately; 3 = Quite a bit; 4 = Extremely). Thus, scores range from 0 to 88. The IES-R is a screening tool rather than a formal diagnostic test, but it correlates well with the diagnosis of PTSD. After reviewing the literature, we chose a cut-off of 33 as the best value to detect a high likelihood of suffering from PTSD. This cut-off provides the highest overall diagnostic power (0.88) with a sensitivity of 0.91, a specificity of 0.82, a positive predictive power of 0.90 and a negative predictive power of 0.84 [15, 19].

The HADS is a valid and simple self-assessment questionnaire to identify and quantify levels of anxiety and depression. It is divided into two subscales: one for assessing symptoms of anxiety (seven items) and the other for evaluating symptoms of depression (seven items). Each item is rated from 0 to 3 points, hence subscale scores range from 0 (no distress) to 21 (maximum distress). Subscale scores of 0 to 7 are considered as normal, whereas scores ≥11 indicate possible anxiety or depression [20]. Subscale scores of 8–10 are considered as borderline abnormal. Our analyses were based on both anxiety and depression subscale scores as well as the total HADS score (the cut-off being the sum of its subscale scores).

Both the IES-R and HADS questionnaires have been translated and validated into the French language and were used as such [21, 22].

### Demographic and clinical characteristics

The demographic characteristics of the participants were collected, as well as information of any past history of trauma. Trauma was defined as being the victim of any event leading to death, risk of death, serious physical injury to oneself or a close relative or a sexual offense. The survey also included questions about any history of personal or familial psychiatric disorders (major depressive disorder, anxiety disorder or previous PTSD).

Participants were also asked if they had provided care to one the victims of the terrorist attacks up to one week after the 13th of November, or if they themselves or one of their close relatives had been a victim or witness of the attacks. If so, they were considered as being exposed to the attacks.

### Statistical analysis

A descriptive statistical analysis was performed. The frequency of symptoms of PTSD, anxiety and depression were calculated for exposed and non-exposed respondents. These features were then compared between the two groups using the Student’s t-test or the Mann-Whitney U test for continuous variables, and the chi-squared test or the Fisher exact test for categorical variables. We specifically used a multinomial regression for the analysis of HADS variables. Statistical significance was defined as *p* < 0.05. Statistical analyses were carried out using R software, version 3.1.3 (R Foundation for Statistical Computing, Vienna, Austria).

## Results

A total of 779 resident physicians (32.3%) responded to the questionnaire. However, 99 of the questionnaires were incomplete, and were thus excluded. Hence, 680 questionnaires (28.2%) were included in the final analysis. The main characteristics of the respondents are summarized in Table 1.

**Table 1:** Characteristics of the study population

*Variables*		Total (*n* = 680)	Exposed (*n* = 216)	Not exposed (*n* = 464)	p
Age *(mean ± SD)*		26.6 (2.0)	26.8 (1.8)	26.5 (2.1)	*0.15*
Gender	*Female (%)*	493 (72.5)	150 (69.4)	343 (73.9)	*0.28*
	*Male (%)*	187 (27.5)	66 (30.6)	121 (26.1)	
Resident seniority (semesters) (mean ± SD*)		5.9 (3.1)	6.7 (3.0)	5.5 (3.0)	*0.01*
Specialty	*Surgery (%)*	71 (10.4)	33 (15.3)	38 (8.2)	
	*Medicine (%)*	310 (45.6)	75 (34.7)	235 (50.6)	
	*Radiology (%)*	37 (4.6)	12 (5.6)	25 (5.4)	
	*Anesthesiology (%)*	89 (13.1)	53 (24.5)	36 (7.8)	
	*Psychiatry (%)*	106 (15.6)	30 (13.9)	76 (16.4)	
	*Pediatrics (%)*	67 (9.9)	13 (6.0)	54 (11.6)	
History of	*Trauma (%)*	103 (15.1)	36 (16.7)	67 (14.4)	*0.72*
	*Personal psychiatric disorder (%)*	104 (15.3)	28 (13.0)	76 (16.4)	*0.30*
	*Familial psychiatric disorder (%)*	287 (42.2)	97 (44.9)	190 (41.0)	*0.39*

*SD Standard Deviation

Among the respondents, 84 (12.4%) were at high risk of having PTSD (IES-R ≥ 33). Anxiety-related symptoms (HADS anxiety score > 10) and depression-related symptoms (HADS depression score > 10) were found in 76 (11.2%) and 16 (2.4%) of the respondents, respectively. In addition 17 (2.5%) of the respondents presented with symptoms of both anxiety and depression. (Table 2).

**Table 2:** Association between psychological characteristics and resident exposure

*Variables*		Total (n = 680)	Exposed (n = 216)	Not exposed (n = 464)	p
HADS	*Abnormal (%)*	17 (2.5)	4 (1.9)	13 (2.8)	*0.79*
	*Borderline abnormal (%)*	58 (8.5)	19 (8.8)	39 (8.4)	
	*Normal (%)*	605 (89.0)	193 (89.3)	412 (88.8)	
	Total score (mean ± SD*)	9.0 (5.4)	9.0 (5.4)	8.9 (5.4)	*0.91*
HADS *Anxiety*	*Abnormal (%)*	76 (11.2)	28 (13.0)	48 (10.3)	*0.67*
	*Borderline abnormal (%)*	150 (22.1)	47 (21.8)	103 (22.2)	
	*Normal (%)*	454 (66.8)	141 (65.2)	313 (67.5)	
	Anxiety score (mean ± SD*)	6.3 (3.4)	6.3 (3.3)	6.3 (3.4)	*0.87*
HADS *Depression*	*Abnormal (%)*	16 (2.4)	6 (2.8)	10 (2.2)	*0.87*
	*Borderline abnormal (%)*	26 (3.8)	9 (4.2)	17 (3.7)	
	*Normal (%)*	638 (93.8)	201 (93.0)	437 (94.1)	
	Depression score (mean ± SD*)	2.7 (2.7)	2.7 (2.7)	2.7 (2.7)	*0.97*
IES-R	*PTSD (%)*	84 (12.4)	40 (18.5)	44 (9.5)	*0.001*
	IES-R (mean ± SD*)	15.6 (13.8)	18.8 (16.6)	14.2 (12.0)	*0.001*
	Intrusion score (mean ± SD*)	7.3 (3.4)	8.7 (7.0)	6.6 (5.4)	*0.001*
	Hypervigilance score (mean ± SD*)	3.4 (4.0)	4.1 (4.7)	3.1 (3.6)	*0.001*
	Avoidance score (mean ± SD*)	5.0 (5.3)	6.1 (6.3)	4.5 (4.8)	*0.001*

*SD Standard Deviation

Of the respondents, 216 (31.8%) were exposed to the Paris terrorist attacks. Forty (18.5%) of the exposed residents were at high risk of having PTSD, compared to 44 (9.5%) non-exposed respondents (*p* = 0.001) (Table 1). Exposure to the terrorist attacks was associated with a higher IES-R score (18.8 ± 16.6 versus 14.2 ± 12.0, *p* = 0.001). No association was found between exposure and symptoms of anxiety or depression*.*

Exposed residents were also found to be significantly more advanced in their residency training: mean of 6.7 (± 3.0) semesters for exposed residents versus 5.5 (± 3.0) semesters for non-exposed (*p* = 0.01). However, residents who presented with PTSD-related symptoms were not significantly younger or less advanced in their residency training than others (26.5 [± 2.1] years old versus 26.6 [± 2.0], *p* = 0.56; and 5.7 [± 3.0] semesters versus 5.9 [± 3.1], *p* = 0.45, respectively).

Of the respondents 493 (72.5%) were female, without any differences between the sexes regarding exposure to the attacks. Significantly more females reported symptoms of PTSD than males (75 [15.2%] versus 9 [4.8%], *p* < 0.001). In addition, exposed females were at higher risk of having PTSD (59/150, 39.3%) compared to non-exposed females (96/343, 28%) (*p* = 0.001).

There was no difference in psychiatric history between the exposed and non-exposed residents (Table 3). But residents who reported PTSD-related symptoms were also significantly more likely to have a history of past trauma than other residents (21/84 [25.0] versus 83/596 [13.9], *p* = 0.004). This was especially significant among non-exposed residents (16/44 [36.4] versus 60/420 [14.3], p = 0.001).

**Table 3:** Association between psychiatric history and PTSD by exposure

		Total (*n* = 680)		p	Exposed (*n* = 216)		p	Not exposed (*n* = 464)		p
PTSD		Yes (*n* = 84)	No (*n* = 596)		Yes (*n* = 40)	No (*n* = 176)		Yes (*n* = 44)	No (*n* = 420)	
History of	*Trauma (%)*	21 (25.0)	83 (13.9)	0.004	5 (12.5)	23 (13.1)	0.92	16 (36.4)	60 (14.3)	0.001
	*Personal psychiatric disorder (%)*	13 (15.5)	90 (15.1)	0.87	7 (17.5)	29 (16.5)	0.91	6 (13.6)	61 (14.5)	0.96
	*Familial psychiatric disorder (%)*	41 (48.8)	246 (41.3)	0.13	17 (42.5)	80 (45.4)	0.73	24 (54.6)	166 (39.5)	0.02

The psychological characteristics of the resident physicians according to the type of exposure are reported in Table 4. The largest HADS and IES-R scores were from those residents who witnessed the attacks or had a close relative among the victims, but they were also from the first responders who took care of the patients at the site of the massacre.

**Table 4:** Psychological characteristics of resident physicians by type of exposure

*Variables*		Victim (*n* = 0)	Witness (*n* = 6)	Relative victim (*n* = 2)	Relative witness (*n* = 6)	First responder (*n* = 8)	Took care of victims the night of the attacks (*n* = 53)	Took care of victims the week following the attacks (*n* = 141)
HADS	*Abnormal (%)*	–	1 (16.7)	1 (50)	0	1 (12.5)	1 (1.9)	0
	*Borderline abnormal (%)*	–	4 (66.6)	1 (50)	1 (16.7)	3 (37.5)	4 (7.5)	6 (4.3)
	*Normal (%)*	–	1 (16.7)	0	5 (83.3)	4 (50)	48 (90.6)	135 (95.7)
	Total score (mean ± SD*)	–	19.5 (6.3)	20.5 (2.1)	14.1 (2.1)	16.1 (2.9)	8.6 (5.2)	8.4 (5.4)
HADS *Anxiety*	*Abnormal (%)*	–	6 (100)	2 (100)	1 (16.7)	4 (57.1)	8 (15.1)	7 (5.0)
	*Borderline abnormal (%)*	–	0	0	4 (66.7)	3 (42.9)	11 (20.8)	29 (20.6)
	*Normal (%)*	–	0	0	1 (16.7)	1 (12.5)	34 (64.1)	105 (78.9)
	Anxiety score (mean ± SD*)	–	13.3 (1.5)	11 (0)	8.8 (1.5)	11.8 (2.4)	6.3 (3.5)	3.6 (3.1)
HADS *Depression*	*Abnormal (%)*	–	2 (33.3)	1 (50)	0	0	1 (1.9)	2 (1.4)
	*Borderline abnormal (%)*	–	1 (16.7)	1 (50)	1 (16.7)	0	2 (3.8)	4 (2.8)
	*Normal (%)*	–	3 (50)	0	5 (83.3)	8 (100)	50 (94.3)	135 (95.8)
	Depression score (mean ± SD*)	–	7.5 (4.9)	10 (1.4)	5.3 (2.4)	4.9 (1.3)	2.4 (2.4)	2.2 (2.2)
IES-R	*PTSD (%)*	–	5 (83.3)	2 (100)	2 (33.3)	5 (62.5)	14 (26.4)	12 (8.5)
	IES-R (mean ± SD*)	–	55.5 (14.7)	52.5 (3.5)	30.5 (9.9)	48.4 (21.1)	22.9 (17.2)	13.3 (11.4)

*SD: Standard Deviation

## Discussion

We documented substantial psychological symptoms among Parisian resident physicians two months after the Paris terrorist attacks. We found that 18.5% of the exposed residents, either those at the scene of the attacks or those caring for the victims, reported symptoms consistent with current PTSD. The most common symptoms were intrusive thoughts, flashbacks and nightmares*.* Comparatively, in a 2015 systematic review Wilson et al. [23] reported that following man-made mass violence 1.3 to 22.0% of first responders presented with probable PTSD. A meta-analysis by Sterud et al. [24] found that the prevalence of PTSD among emergency personnel in Western European countries ranged from 15 to 21.5%. Our results are within the higher end of this range. However, the IES-R cut-off value used by Sterud et al. was lower than ours (20 compared to 33), we chose this cut-off in order to have a higher specificity. Another systematic review found that the prevalence of PTSD in rescue workers worldwide is approximately 10% [25]. In our study, exposed residents were significantly more likely to report symptoms consistent with current PTSD than non-exposed residents (18.5% versus 9.5%), highlighting the psychological impact of being confronted by disaster. In comparison, the prevalence of PTSD in the general population of Western Europe is estimated to be 1.1% [26]. To the best of our knowledge there are no studies on the prevalence of PTSD among the general population of France following the 2015 terrorist attacks with which to compare our results. A study conducted in the United States, one to two months following the events of September 11, found a prevalence of probable PTSD of 11.2% among the general population in the New York City metropolitan area and 4.0% in the rest of the country.

In our study, women were at a higher risk of developing PTSD than men, especially when they had been exposed to the terrorist attacks. This result is consistent with the literature [27] and is significant, as PTSD persists longer in women, particularly after exposure to inter-personal trauma.

The increasing waves of civilian attacks since the 1980s have drawn urgent attention to the need for structured psychological care for victims [28, 29]. Furthermore, the incidence of PTSD has been reported to be higher for survivors of terrorist attacks than for survivors of other traumatic situations such as motor vehicle accidents or natural disasters [30]. Inter-personal traumas are more likely to cause PTSD than indirect traumas [3]. Among inter-personal traumas, inter-personal violence is most likely to cause PTSD (20.9%) in both victims and relatives [31, 32].

No difference in the prevalence of anxiety or depression was observed between respondents who were exposed to the attack and those who were not. Of all the respondents, 11.2% reported symptoms consistent with an anxiety disorder, and 22.1% had symptoms suggesting a borderline anxiety disorder (with a HADS anxiety subscale score of between 8 and 10). This is considerably higher than the 2.1% of anxiety disorders recently reported in the general population of France [33], and could suggest an underlying abnormal anxiety rate among our participants. This is concerning as anxiety leads to a decreased attention span and a reduction in cognitive abilities. Of the respondents, 2.4% had a probable diagnosis of depression and 6.4% had symptoms of depression. Mata et al. performed a meta-analysis [7] of symptoms of depression among resident physicians, and although they also used the HADS scale they found the prevalence of depression to be higher than in our study (15%). By comparison, the prevalence of depressive disorders in the general population of France is 6.0% [33]. In addition, we found no difference in depression or anxiety symptomatology between respondents from the different medical and surgical specialties.

We also found that exposed residents were significantly more advanced in their training. This result is interesting but difficult to interpret. Our first hypothesis would be that they volunteered to be on the medical teams, rather than exposing the younger residents. Another hypothesis would be that the medical teams in charge of caring for the victims were composed of more advanced residents because of how the residency program is organized in France. However, it is also possible that a higher proportion of advanced exposed residents may have responded to the questionnaire, with less advanced exposed residents avoiding answering it, possibly because of post-traumatic stress. Whatever the hypothesis, this difference makes us fear an underestimation of the frequency of PTSD among exposed residents, especially since the risk of suffering from a mental illness is inversely proportional to age [8], whatever the cause of the distress.

Our decision to carry out this survey two months after the attacks was motivated by several reasons: First and foremost, we wanted to observe a respectful delay. Secondly, the diagnostic criteria for PTSD as stated in the DSM 4 and 5 [34] requires symptoms that persist for more than one month. When symptoms last for more than a month, remission can take a long time. There is no clear published data on the rate of remission of PTSD during the second month after a trauma, but it has been reported that a third of PTSD sufferers will be in remission by six months [32], and that the median time to remission with appropriate treatment is 36 months [35]. Finally, our aim was to not focus on immediate symptoms of depression, anxiety or PTSD, which are frequent and mostly temporary, but to assess persistent PTSD, anxiety or depression. Persistent symptoms can induce cognitive deficits, especially in executive functions, the speed of information processing, attention and memory [36], which can significantly impact the performance of a resident physician. To our knowledge, no study has explored the link between the psychological consequences of trauma and medical errors. De Oliveira et al. [37] have shown a strong association between the presence of psychological suffering and multiple medical errors among anesthesiology trainees. If patients are the first victims of medical error, then physicians are termed the “second victims” as they often experience feelings of distress, guilt, shame and depression in response. [38, 39]. In future research endeavors, repetition of the survey at different time points would make it possible to assess the progression of symptoms of PTSD, anxiety and depression.

Several limitations should be considered when interpreting the findings of this study.

First, even though our participation rate was of 32.3% across all residency specialties, with 680 (28.2%) complete questionnaires, non-response bias is a concern. It is possible that the resident physicians who were dealing with the worst implications of the attacks did not have the ability to participate. In addition, residents which are familiar with screening measures might have under-reported their symptoms. This would have made us underestimate post-traumatic distress among residents. On the other hand, it is also possible that residents who were unaffected by the attacks felt less invested and thus were less willing to participate. Hence, it is difficult to estimate how representative these responses are of the overall group of residents or how biased the results are due to non-responses. Secondly, data were collected through an online self-administered questionnaire, which is subject to selection and measurement bias. However, residents are trained to perform self-administered questionnaires, due to their frequent use during clinical practice and scientific research. Most of them, including non-psychiatry residents, are aware of the mental illnesses presently studied. It is also unlikely that assessments were under-quoted thanks to the anonymity of the questionnaire. In order to evaluate persistent disorders, the questions focused on symptoms ongoing over seven days. We therefore presume that the reliability of the responses is high. We did not collect data related to the psychological support that the resident physicians may have accessed since the attack, which may have impacted our estimation of the rate of PTSD among our subjects. Data in the literature on very early interventions (such as debriefings) or early interventions (first-month psychotherapies) for the treatment of acute stress disorders are divergent and do not allow a gold standard of care to be defined. Reviews suggest that debriefings may not have any positive effects [40] and may even have a long-term detrimental effect [41]. On the other hand, psychotherapeutic interventions for the treatment of PTSD are highly effective. The NICE guidelines for PTSD [42] are still valid. They recommend that PTSD sufferers should be offered a course of trauma-focused psychological treatment (trauma-focused cognitive behavioral therapy or eye movement desensitization and reprocessing). In the medical field, supervision by senior physicians could have a positive effect on residents suffering from PTSD, providing support, gradual empowerment and a sense that their work is valued [43].

Our findings highlight the need to implement continuous measures to prevent PTSD and anxiety in resident physicians, as well as educational campaigns about PTSD and its treatment options. We believe that after an event residency programs should offer their residents systematic screening for PTSD, periodically monitor them for psychological consequences and ensure access to appropriate care for those affected.

## Conclusions

Overall, we found that the frequency of symptoms of mental distress among our subjects was high, with 12.4% of residents suffering from PTSD and 11.2% from symptoms of anxiety. Critical incidents can adversely impact the mental well-being of medical professionals. In addition to effects on the mental health of resident physicians, their PTSD, depression and anxiety may also affect patient care and safety.

Dedicated screening and care strategies must be considered in the event of new attacks, based on established knowledge for the management of PTSD. In the future it would be beneficial to evaluate the psychological condition of resident physicians at different time points.

## Additional file

Additional file 1: Questionnaire sent to the physician residents *(Translated from the French version used for the survey)*. (DOCX 28 kb)

## Abbreviations

APHP: Assistance-Publique des Hôpitaux de Paris
DSM: Diagnostic and Statistical Manual of Mental Disorders
HADS: Hospital Anxiety Depression Scale
IES-R: Impact of Event Scale– Revised
PTSD: Post-traumatic stress disorder
SIHP: Syndicat des Internes des Hôpitaux de Paris
