# Supplementary material for: The impact of the Paris terrorist attacks on the mental health of resident physicians
Source: BMC Psychiatry. 2019 Feb 21;19:79. doi: 10.1186/s12888-019-2058-y (PMC6385411; doi:10.1186/s12888-019-2058-y)
Supplement: Supplementary file 1 — Questionnaire sent to the physician residents (Translated from the French version used for the survey). (DOCX 28 kb) [file 12888_2019_2058_MOESM1_ESM.docx]

**Supplementary file.**

**Questionnaire sent to the physician residents *(Translated from the French version used for the survey).***

1. Background information

A1 - What is your age (years)?

A2 - How many semesters of residency have you completed?

A3 - Gender?

- Female
- Male
- Other

A4 - What is your specialty?

- Surgery
- Medicine
- Radiology
- Anesthesiology
- Psychiatry
- Pediatrics

A5 - Do you have any past history of trauma?

*Trauma defined as being the victim of any event leading to death, risk of death, serious physical injury to oneself or a close relative or a sexual offense.*

- Yes
- No

A6 - Do you have a psychiatric history (major depressive disorder, anxiety disorder or other)?

- Yes
- No

A7 - Do you have a family psychiatric history (major depressive disorder, anxiety disorder or other)?

- Yes
- No

A8 - Have you been personally affected by the attacks of 13 November 2015?

- I was one of the victims
- I witnessed the attack
- A close relative of mine was one of the victims
- I was one of the first caregivers on site of the attack
- I took care of victim(s) the night of the attacks
- I took care of victim(s) the week following the attacks

1. Hospital Anxiety and Depression Scale (HADS) (1).

*The following questions relate to your current status.*

B1 – I feel tense or “wound up”:

- Most of the time
- A lot of time
- From time to time, occasionally
- Not at all

B2 – I still enjoy the things I used to enjoy:

- Definitely as much
- Not quite so much
- Only a little
- Hardly at all

B3 - I get a sort of frightened feeling as if something awful is about to happen:

- Very definitely and quite badly
- Yes, but not too badly
- A little, but it doesn't worry me
- Not at all

B4 - I can laugh and see the funny side of things:

- As much as I always could
- Not quite so much now
- Definitely not so much now
- Not at all

B5 - Worrying thoughts go through my mind:

- A great deal of the time
- A lot of the time
- From time to time but not too often
- Only occasionally

B6 - I feel cheerful:

- Not at all
- Not often
- Sometimes
- Most of the time

B7 - I can sit at ease and feel relaxed:

- Definitely
- Usually
- Not often
- Not at all

B8 - I feel as if I am slowed down:

- Nearly all the time
- Very often
- Sometimes
- Not at all

B9 - I get a sort of frightened feeling like 'butterflies' in the stomach:

- Not at all
- Occasionally
- Quite often
- Very often

B10 - I have lost interest in my appearance:

- Definitely
- I don't take so much care as I should
- I may not take quite as much care
- I take just as much care as ever

B11 - I feel restless as if I have to be on the move:

- Very much indeed
- Quite a lot
- Not very much
- Not at all

B12 - I look forward with enjoyment to things:

- As much as ever I did
- Rather less than I used to
- Definitely less than I used to
- Hardly at all

B13 - I get sudden feelings of panic:

- Very often indeed
- Quite often
- Not very often
- Not at all

B14 - I can enjoy a good book or radio or TV program:

- Often
- Sometimes
- Not often
- Very seldom

1. Impact of Event Scale- Revised (IES-R) (2)

*Below is a list of difficulties people sometimes have after stressful life events. Please read each item, and then indicate how distressing each difficulty has been for you DURING THE PAST SEVEN DAYS with respect to the Paris terrorist attacks which occurred on November 13^th^, 2015. How much were you distressed or bothered by these difficulties?*

C1 - Any reminder brought back feelings about it:

- Not at all
- A little bit
- Moderately
- Quite a bit
- Extremely

C2 - I had trouble staying asleep:

- Not at all
- A little bit
- Moderately
- Quite a bit

C3 - Other things kept making me think about it:

- Not at all
- A little bit
- Moderately
- Quite a bit

C4 - I felt irritable and angry.

- Not at all
- A little bit
- Moderately
- Quite a bit

C5 - I avoided letting myself get upset when I thought about it or was reminded of it:

- Not at all
- A little bit
- Moderately
- Quite a bit

C6 - I thought about it when I didn’t mean to:

- Not at all
- A little bit
- Moderately
- Quite a bit

C7 - I felt as if it hadn’t happened or wasn’t real:

- Not at all
- A little bit
- Moderately
- Quite a bit

C8 - I stayed away from reminders of it:

- Not at all
- A little bit
- Moderately
- Quite a bit

C9 - Pictures about it popped into my mind:

- Not at all
- A little bit
- Moderately
- Quite a bit

C10 – I was jumpy and easily startled:

- Not at all
- A little bit
- Moderately
- Quite a bit

C11 – I tried not think about it:

- Not at all
- A little bit
- Moderately
- Quite a bit

C12 – I was aware that I still had a lot of feelings about it, but I didn’t deal with them:

- Not at all
- A little bit
- Moderately
- Quite a bit

C13 - My feelings about it were kind of numb:

- Not at all
- A little bit
- Moderately
- Quite a bit

C14 - I found myself acting or feeling like I was back at that time:

- Not at all
- A little bit
- Moderately
- Quite a bit

C15 - I had trouble falling asleep:

- Not at all
- A little bit
- Moderately
- Quite a bit

C16 - I had waves of strong feelings about it:

- Not at all
- A little bit
- Moderately
- Quite a bit

C17 - I tried to remove it from my memory:

- Not at all
- A little bit
- Moderately
- Quite a bit

C18 - I had trouble concentrating:

- Not at all
- A little bit
- Moderately
- Quite a bit

C19 - Reminders of it caused me to have physical reactions, such as sweating, trouble breathing, nausea, or a pounding heart:

- Not at all
- A little bit
- Moderately
- Quite a bit

C20 - I had dreams about it:

- Not at all
- A little bit
- Moderately
- Quite a bit

C21 - I felt watchful and on-guard:

- Not at all
- A little bit
- Moderately
- Quite a bit

C22 - I tried not to talk about it:

- Not at all
- A little bit
- Moderately
- Quite a bit

1. Zigmond AS, Snaith RP. The hospital anxiety and depression scale. Acta psychiatrica Scandinavica. 1983;67(6):361-70.

2. Creamer M, Bell R, Failla S. Psychometric properties of the Impact of Event Scale - Revised. Behaviour research and therapy. 2003;41(12):1489-96.
